# Microalgae Xanthophylls: From Biosynthesis Pathway and Production Techniques to Encapsulation Development

**DOI:** 10.3390/foods10112835

**Published:** 2021-11-17

**Authors:** Slim Smaoui, Mohamed Barkallah, Hajer Ben Hlima, Imen Fendri, Amin Mousavi Khaneghah, Philippe Michaud, Slim Abdelkafi

**Affiliations:** 1 Laboratoire de Microorganismes et de Biomolécules, Centre de Biotechnologie de Sfax, Route Sidi Mansour Km 6 B.P. 117, Sfax 3018, Tunisia; slim.smaoui@yahoo.fr; 2 Laboratoire de Génie Enzymatique et Microbiologie, Equipe de Biotechnologie des Algues, Ecole Nationale d’Ingénieurs de Sfax, Université de Sfax, Sfax 3038, Tunisia; mohamedbarkallah@gmail.com (M.B.); hajer_benhlima@yahoo.fr (H.B.H.); 3 Laboratoire de Biotechnologie Végétale Appliquée à l’Amélioration des Cultures, Faculté des Sciences de Sfax, Université de Sfax, Sfax 3038, Tunisia; imen.fendri@fss.usf.tn; 4 Department of Food Science and Nutrition, Faculty of Food Engineering, University of Campinas (UNICAMP), Campinas 13083-862, SP, Brazil; mousavi.amin@gmail.com; 5 Institut Pascal, Université Clermont Auvergne, CNRS, Clermont Auvergne INP, F-63000 Clermont-Ferrand, France

**Keywords:** xanthophylls, microalgae, biosynthesis, processes, extraction, encapsulation

## Abstract

In the last 20 years, xanthophylls from microalgae have gained increased scientific and industrial interests. This review highlights the essential issues that concern this class of high value compounds. Firstly, their chemical diversity as the producer microorganisms was detailed. Then, the use of conventional and innovative extraction techniques was discussed. Upgraded knowledge on the biosynthetic pathway of the main xanthophylls produced by photosynthetic microorganisms was reviewed in depth, providing new insightful ideas, clarifying the function of these active biomolecules. In addition, the recent advances in encapsulation techniques of astaxanthin and fucoxanthin, such as spray and freeze drying, gelation, emulsification and coacervation were updated. Providing information about these topics and their applications and advances could be a help to students and young researchers who are interested in chemical and metabolic engineering, chemistry and natural products communities to approach the complex thematic of xanthophylls.

## 1. Introduction

Microalgae and cyanobacteria are a highly diverse group of photoautotrophs. These microorganisms are present across all aquatic environments [1]. Common microalgae ancestors lived in an aquatic environment approximately 3 billion years ago and in this period, microalgae evolved and diversified [2]. Nowadays, microalgae and cyanobacteria are listed in 72,000 species and 16 classes [3]. The largest algae groups are green algae (*Chlorophyceae*), diatoms (*Bacillariophyceae*) and golden algae (*Chrysophyceae*). These microorganisms occupy the base of the aquatic food chain. Some are adapted to growth under a broad spectrum of environmental stressors including cold, heat, drought, salinity, anaerobiosis and osmotic pressure [4]. They have the intrinsic capacity to fix dioxide (CO_2_) with the aid of sunlight, and notably contribute to the production of oxygen on earth by photosynthesis. Besides their essential role in ecosystems, cyanobacteria and microalgae are being exploited commercially thanks to their richness in bioactive health beneficial compounds such as polysaccharides, proteins, pigments (chlorophylls, carotenoids and phycobiliproteins), lipids (including oils and polyunsaturated fatty acids) and vitamins [5,6,7,8,9,10]. Geographically, the principal producers of commercial microalgae biomass are in the United States, Taiwan, China, Japan, Spain, Brazil and Germany, comprising an annual production of 19,500 tons of biomass, and generating global revenue of about 5.8 billion USD [11]. Among the large diversity of high-value added compounds from microalgae, carotenoids are one of the most pertinent groups to be valorized. The anti-oxidants α-carotene, β-carotene, lycopene, astaxanthin (ASX), lutein and canthaxanthin are the main high value carotenoids from microalgae. Chemically, carotenoids have a general C40 backbone structure composed of isoprene units (terpenoid), characterized by a color that turns from yellow to red [12].

These pigments are classified into carotenes (do not containing O_2_) and xanthophylls (containing O_2_) [13]. Xanthophylls have received a lot of attention because of their diverse biological functions in all living organisms [14,15]. Their most interesting biological roles are associated with their antioxidant properties, depending on their molecular structure [16]. Xanthophylls play the role of potent scavengers of reactive oxygen (ROS) and reactive nitrogen (RNS) species and effective chain-breaking antioxidants [17]. The addition of xanthophylls in foods is very beneficial as they are able to protect cells against oxidative damage [18]. They are also of interest because they protect the quality of food products during processing and storage. Among marine xanthophylls, the most interesting and abundant is fucoxanthin (C_42_H_58_O_6_), accounting for about 10% of total carotenoid production [19]. High concentrations of this xanthophyll are found in the chloroplasts of several brown algae, giving them an olive-green or brown color, and in diatoms (*Bacillariophyta*) [20,21]. The second xanthophyll of interest is ASX (C_40_H_52_O_4_), which is a red-pink carotenoid. It is known as a powerful antioxidant since it has about ten times more antioxidant activity than other carotenoids [11]. The principal natural source of this xanthophyll is the microalgae *Haematococcus pluvialis*, which is already produced on an industrial scale [20]. Lutein and zeaxanthin (C_40_H_56_O_2_), two isomers, are yellow pigments found in several microalga species such as *Chlorella minutissima* and *Nannochloropsis oculata*. The cyanobacteria, *Spirulina platensis* pacifica is also a relevant source of the red β-cryptoxanthin (C_40_H_56_O) and zeaxanthin. Today, the most available pigments on the market are fucoxanthin and ASX, followed by lutein (C_40_H_52_O_2_), a yellow isomer of zeaxanthin and canthaxanthin (C_40_H_52_O_2_) from green algae [5,22]. Up to now, most commercial xanthophylls have been produced artificially [16]. However, interest in natural foods has increased the demand for natural sources of xanthophylls [23].

Microalgae are a sustainable origin for xanthophyll production and have numerous benefits in comparison to many other natural sources. In order to obtain high concentrations of specific xanthophylls, some environmental stresses and culture conditions can be applied to modulate the biochemical composition of microalgae [24]. However, under basic growth conditions, the concentration of produced xanthophylls is usually too low, making the production of carotenoids from microalgae economically unprofitable [25]. To improve its economic profitability, it is necessary to explore the metabolic pathways and to understand how environmental factors and the integration of nutrients into microalgae cultures affect the production of xanthophylls. 

Today, there is a great deal of interest in investigating the beneficial effects of the major xanthophylls in the human diet through their use as feed additives, dietary supplements and food colorants in several sorts of food. In this review, we will first describe the chemical structures of the principal commercial xanthophylls and their different synthetic pathways in microalgae. Secondly, we will analyze the important strategies implemented to optimize their biotechnological production, both by the manipulation of the culture conditions as well as by genetic engineering. In particular, this review suitably details the recent advances in the use of new technologies to recover xanthophylls from microalgae. Finally, we will describe the current encapsulation processes of xanthophylls and their effects on their bioactive properties when used as food ingredients.

## 2. Main Xanthophylls Present in Microalgae

Microalgae present a raw material of interest because of their pigment content, which is known by their biological activities. Currently, few species are used to produce xanthophylls, as their industrial exploitation is rare. Table 1 illustrates the current use of microalgae in the field of xanthophyll production. It includes data about the major microalgae species producing xanthophylls, the applications and the principal extraction processes used to purify these molecules.

### 2.1. Fucoxanthin

Fucoxanthin, a secondary metabolite, is produced in the chloroplasts of brown microalgae and diatoms, giving them an olive-green or brown color [16]. This xanthophyll represents up to 10% of the global production of carotenoids in the aquatic environments [62]. The unique structure of fucoxanthin is based on an unusual allenic bond, a conjugated carbonyl group in the polyene chain and an epoxide(Table 2), at the origin of its antioxidant properties. However, the difference is that fucoxanthin presents antioxidant attributes even under anoxic conditions, whereas the other xanthophylls exhibit virtually no quenching capacity under those conditions. Consequently, fucoxanthin may be performing key roles in tissues which have a low level of oxygen. Whatever the dose used, fucoxanthin does not present toxicity and mutagenicity under experimental conditions [62]. Many bioactivities have been reposted regarding fucoxanthin. Several articles have been published about its antioxidant, anticancer, anti-inflammatory, antimicrobial, and antihypertensive, anti-obesity, anti-diabetic, and anti-angiogenic activities, as well as its photoprotective and neuroprotective effects (Table 1) [63,64,65,66,67,68]. Considering all these properties, fucoxanthin has an important potential for applications in different domains, from supplemented foods and complements, to pharmaceutical drugs and anti-aging cosmetics for all pathologies including cancer. For these reasons, the fucoxanthin market is expected to keep growing and reach 120 million dollars by 2022 [69]. 

The worldwide production of fucoxanthin was about 500 t in 2016 and was projected to grow at an annual rate of 5% up to 2021 [69]. In 2017, Galasso et al. reported that there are principal industrially produced fucoxanthin as a Nutraceutical and cosmeceutical applications and both of the industries were in China, such as Leili Natural Products Co., Ltd. (Kibbutz Ketura, Israel) and AlgaNova International [13]. In Israel, Algatechnologies, Ltd. (Kibbutz Ketura, Israel) (“Algatech”) launches Fucovital^®^, a patented naturel fucoxanthin oleoresin produced and extracted from microalgae.

### 2.2. Astaxanthin (ASX)

ASX is a secondary keto-carotenoid with a pink-color containing two additional oxygenated groups on each ring compared to other carotenoids. It is 550 and 11 times more effective as a singlet oxygen scavenger than vitamin E and β-carotene, respectively due to its ability to bind to the cell membrane [70]. Due to its spectacular antioxidant properties, ASX shows higher anti-aging and anti-inflammatory activities than other carotenoids [71]. This xanthophyll occurs naturally in a large variety of microalgae such as *Haematococcus pluvialis* and *Chlorella zofingiensis* [72]. *Haematococcus pluvialis* is the most widely used natural source for the industrial production of this pigment with yields of up to 3.8% DW [32,33,73]. It is relatively easy to purify ASX from microalgae because it represents 90% of the total carotenoid content [74]. For all these reasons, various products containing ASX are already available on the international market in diver forms such as soft, oils, syrups, creams, capsules with a market value of USD 1.0 billion in 2019 [16]. Two examples are AstaPure^®^ (Algatech Ltd., Kibbutz Ketura D.N, Israel, https://www.algatech.com/, accessed on 15 April 2021) and BioAstin (Cynotech Corporation, Kailua-Kona, HI, USA, https://www.Cyanotech.com/, accessed on 15 April 2021) produced from the microalgae *Haematococcus pluvialis*.

### 2.3. Lutein

Lutein is one of the two essential compounds of the macular pigment of the retina [75]. This xanthophyll is synthesized only by algae, is abundant in green microalgae [16]. It acts as a strong antioxidant able to filter phototoxic blue-light radiation [76]. *Chlorella* is an effective source of lutein and a good candidate for its production [43,77]. The results of these studies showed that nitrogen limitation and high temperature stress have been identified as the main parameters impacting lutein accumulation. However, the cultivation conditions of other microalgae species with significant lutein contents such as *Scenedesmus almeriensis*, *Dunaliella salina* and *Galdieria sulphuraria* have also been an optimized production of this pigment. According to the results of these optimizations, the contribution of nutrients has a lesser effect due to the high tolerance of these microalgae to large ranges of salinity, pH, temperature and nutrient concentration [78,79].

Up to now, there are no production systems for the commercial production of lutein from microalgae. Outdoor production systems of *Muriellopsis* sp have been installed at a pilot scale. In it *Muriellopsis* sp. was grown in 50 L tubular PBRs which produced 40 g/m^2^/d of lutein. *Scenedesmus almeriensis* biomass wasproduced in a 4000 L tubular PBR for lutein production and 290 mg/m^2^/d of lutein was obtained [79]. The lutein market is expected to reach USD 410 million in 2027 at a compound annual growth rate of 6.1% over the planned period 2020–2027.

### 2.4. Zeaxanthin

Zeaxanthin is present in large quantities in plants and algae. It plays a major role in the xanthophyll cycle. Zeaxanthin is a structural isomer of lutein, and the two xanthophylls differ from each other only in the location of a single double bond. Indeed, in zeaxanthin, all the double bonds are conjugated. This pigment performs an essential role in the prevention of age-associated macular degeneration; one of the major blindness causes [80]. Furthermore, this compound may also be used in cancer prevention via its powerful anti-inflammatory effect [81]. For these reasons, its extraction from algae, microalgae and cyanobacteria is of great interest. There are several studies that have investigated the production of this pigment from some microalgae species. Among them, one publication describes a genetically modified strain of *Nannochloropsis oculata*, which accumulates 13 mg of zeaxanthin per gram of dried biomass [51]. Other species synthetizing zeaxanthin include *Chlorella ellipsoidea* and *Synechocystis* sp. (Table 1). These microalgae can accumulate zeaxanthin up to nine times higher than red peppers which are the traditional source of this pigment. In addition, microalgae have the advantage over plants that the zeaxanthin is present in a free form, while it is present as a monoester and a diester of zeaxanthin in plants [52]. For this reason, numerous works focused on processes to produce zeaxanthin at a large scale from microalgae [81]. 

### 2.5. Violaxanthin

Violaxanthin is an orange xanthophyll pigment. It is present in diverse groups of microalgae (Table 1). This xanthophyll is biosynthesized through epoxidation of zeaxanthin. In total, there are a few studies describing the isolation of violaxanthin from *Dunaliella tertiolecta* [82], *Chlorella ellipsodea* and *Chlorella vulgaris* as sources. There are still additional microalgae genera that could be used to produce this xanthophyll and to broaden its applications. 

### 2.6. Canthaxanthin

Canthaxanthin is a diketo-carotenoid with an orange-red color. For several green microalgae and cyanobacteria this secondary metabolite is produced at the end of the growth phase in addition to the primary ones [17]. It is used as a food colorant (E161g) in the United States and certain countries in Europe. Canthaxanthin is biosynthesized through the action of β-carotene ketolase, which catalyzes addition of carbonyl groups at the 4 and 4′ positions of β-carotene. The regulation of canthaxanthin biosynthesis has been studied recently in *Haematococcus pluvialis* in order to improve its large-scale production [83]. The canthaxanthin content in the transformed cells was found to be 8–10-fold higher in transformed cells compared to the non-transformed (NT) *Haematococcus pluvialis*.

### 2.7. β-Cryptoxanthin

β-cryptoxanthin is a carotenoid with a similar chemical structure, but is more polar than β-carotene. This pro-vitamin A is oxidized and isomerized in the presence of light [81]. It is used as a food colorant in certain countries and is designed as E161c. This pigment is much less abundant than β-carotene, and it can only be found in some fruits and vegetables like tangerines and pumpkin [84,85]. It is also possible to find this compound in *Spirulina platensis* and *Pandorina morum* or *Nanochlorum eucaryotum* (Table 1). According to several studies, β-cryptoxanthin protects against many diseases due to its antioxidant and anti-inflammatory activities [14,84]. 

### 2.8. Diatoxanthin

Diatoxanthin is a xanthophyll found in diatoms. These microorganisms are often called golden brown microalgae, due to their content of xanthophylls, including diatoxanthin, fucoxanthin and diadinoxanthin [86]. Diatoxanthin has the function of protection system against light saturation. Due to its presence, the microalgae are capable of rapidly acclimatizing to the differences in the quantity of light received and therefore continue to realize their vital functions without alterations [87]. Thus, a valid way to enhance the production and performance of this xanthophyll is to increase the blue-light irradiation (300 μmol photons/m^2^/s), especially for *Euglena gracilis* [88].

### 2.9. Diadinoxanthin

Diadinoxanthin was found only in diatoms and other limited microalgal groups. This pigment might be considered as a diatom-specific xanthophyll [61]. Diadinoxanthin, together with fucoxanthin, can be obtained from neoxanthin after isomerization of one of its allenic double bonds [89]. Its antioxidant activity is due to its ability to trap singlet oxygen, which protects cells against oxidative damage [90].

**Table 2:** Common names, IUPAC nomenclatures, molecular formulas and chemical structures of the most commercialized xanthophylls.

Common Names	IUPAC Nomenclature	Molecular Formulas	Chemical Structures	References
**Fucoxanthin**	3,5′-Dihydroxy-8-oxo-6′,7′-didehydro-5,6-epoxy-5,6,7,8,5′,6′-hexahydro-β,β-caroten-3′-yl acetate	C_42_H_58_O_6_	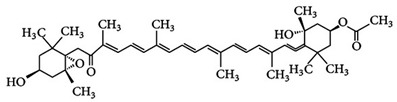 3*S*,5*R*,6*S*,3′*S’*,5′*R*,6′*R*)-3,5′-dihydroxy-8-oxo-6′,7′-didehydro-5,6-epoxy-5,6,7,8,5′,6′-hexahydro-β,β-caroten-3′-yl acetate	[91]
**ASX**	3,3′-Dihydroxy-β,β-carotene-4,4′-dione	C_40_H_52_O_4_	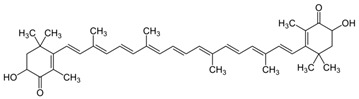 (3*S*,3*0S*)-3,3*0*-dihydroxy-β,β-carotene-4,4*0*-dione	[12]
**Lutein**	β- ε-Carotene-3,3′-diol	C_40_H_56_O_2_	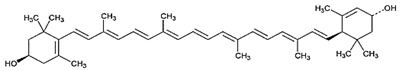 (3*R*,3′*R*,6′*R*)-β,ε-carotene-3,3′-diol	[92]
**Zeaxanthin**	β,β-Carotene-3,3′-diol	C_40_H_56_O_2_	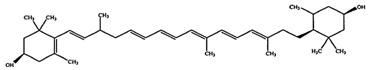 (3*R*,3*0R*)-β,β-carotene-3,3′-diol	[92]
**Violaxanthin**	5,5′,6,6′-Tetrahydro-5,6:5′,6′-diepoxy-β,β-carotene-3,3′-diol	C_40_H_56_O_4_	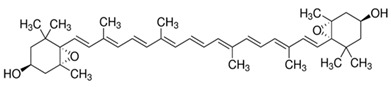 *S*,3′*S*,5*R*,5′*R*,6*S*,6′*S*)-5,5′,6,6′-tetrahydro-5,6:5′,6′-diepoxy-β,β-carotene-3	[93]
**Canthaxanthin**	β,β-Carotene-4,4*0*-dione	C_40_H_52_O_2_	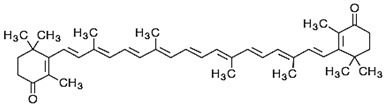 *trans-*β-carotene-4,4′-dione	[94]
**β-Cryptoxanthin**	β,β-Caroten-3-ol	C_40_H_56_O	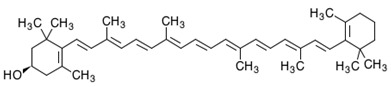 (3*R*)-β,β-Caroten-3-ol	[95]
**Diadinoxanthin**	5,6-Epoxy-7’,8’-didehydro-5,6-dihydro-b,b-carotene-3,3-diol	C_40_H_54_O_3_	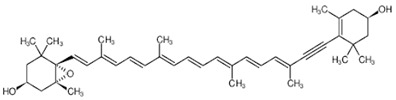 (3*S*,3’*R*,5*R*,6*R*)-7’,8’-Didehydro-3,6-epoxy-5,6-dihydro- β, β -carotene-3’,5-diol	[61]
**Diatoxanthin**	3,3′-7,8-Didehydro-ß,ß-carotene-3,3’-diol	C_40_H_54_O_2_	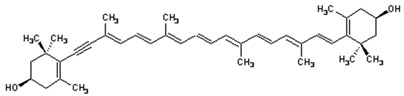 (3*R*,3’*R*)-7,8-Didehydro- β, β -carotene-. 3,3’-diol	[61]

## 3. Structures of Xanthophylls

Carotenoids are the most diverse and widespread lipophilic compounds found in nature and are generally colored yellow, orange or red [96]. Most carotenoids share a common C40 backbone structure of isoprene units (termed terpenoid) and are divided into two groups: carotenes and xanthophylls. Each of the carotenoids consist of different trans and cis isomers. Xanthophylls are oxidized derivatives of carotenes (which are hydrocarbons). The molecules most representative of xanthophylls are lutein, fucoxanthin, β-cryptoxanthin, ASX and zeaxanthin. They are more polar than carotenes due to the presence of oxygen at the ends of their rings in the form of a methoxy, keto, hydroxy, epoxy and carboxy groups [5]. Indeed, lutein and zeaxanthin are characterized by the presence of -OH groups in their structures, while canthaxanthin and echinenone contain = O groups. ASX is a xanthophyll which has both -OH and =O groups in its structure. There are xanthophylls such as violaxanthin and diadinoxanthin, which contain epoxy groups in their structures, while others such as dinoxanthin and fucoxanthin have acetyl groups. In addition, the two acetyl-containing carotenoids also contain the group C=C=C (allene), which is exceptional in natural products [97]. Certain carotenoids such as hetero-, allo-, diadino-, diato-, pyro- croco- and monadoxanthine present in their structures C≡C (acetylene) groups. As powerful antioxidants, xanthophylls are generally sensitive to several factors such as light, oxygen and heat, leading to difficulties in their purification and storage. The structures of the most abundant xanthophylls are shown in Table 2.

## 4. Biosynthesis of Xanthophylls

**Figure 1:**
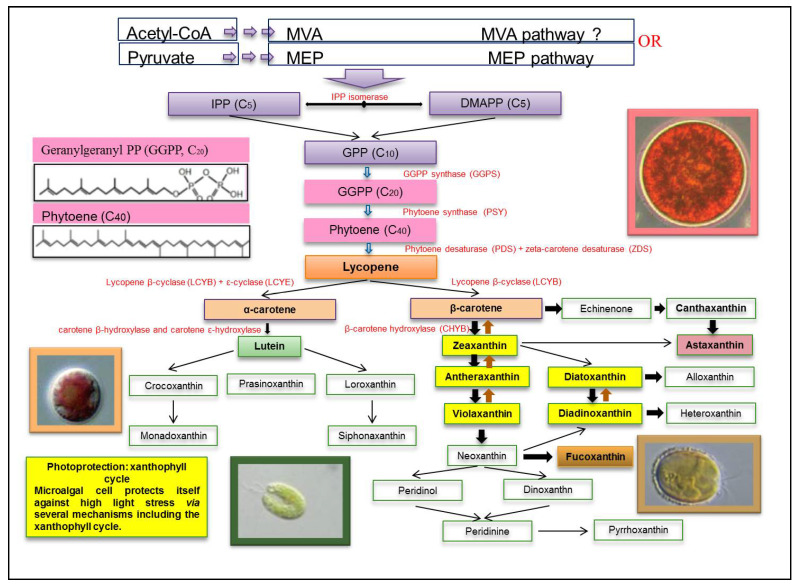
General pattern of xanthophylls synthesis in microalgae. The implicated enzymes in each step of biochemical conversion are listed in red. Their corresponding genes are noted in parentheses. The microalgae *Porphyridium* sp. (orange border), *Tetraselmis* sp. (green border), *Pleurochrysis* (brown border) and *Haemotococcus* sp. (pink border) are presented as principal producers of β-carotene, lutein, fucoxanthin and ASX, respectively.

All carotenoids are synthetized from a common precursor to all isoprenoids, the isopentenyl pyrophosphate (IPP). The biosynthesis of IPP may generally be performed by one of two different pathways (Figure 1): the mevalonate pathway (MVA) in euglenophytes and the plastidial 2-C-methyl-D-erythriol-4-phosphate (MEP) pathway in Chlorophyceae and Cyanophyceae [42,67,96,98]. The MEP pathway is considered to be the main biosynthetic pathway of carotenoids in several microalgae species such as *Chlorella vulgaris*, *Dunaliella salina*, *Scenedesmus* sp., and *Haematococcus pluvialis* [98,99]. In this pathway, the carotenoid biosynthesis begins through IPP, which isomerises to dimethylallyl pyrophosphate (DMAPP) by the action of the IPP isomerase. Elongation of the carbon chain took place through continuous head-to-tail condensation of IPP to DMAPP followed by growing of the polyprenyl pyrophosphate chain by the action of the prenyltransferase [55,100]. The second committing step in carotenoid biosynthesis is the condensation of two molecules of geranyl pyrophosphate (GPP, C10), catalyzed by the GGPP synthase, to yield geranylgeranyl PP (GGPP, C20) [42]. After that, the colourless C40 carotenoid phytoene is formed through the condensation of two GGPP (C20) molecules by the action of phytoene synthase (PSY) [42]. From this step onwards, phytoene undergoes a series of sequential desaturations (four desaturation steps) catalyzed by phytoene desaturase (PDS) and zeta-carotene desaturase (ZDS)leading to the formation of pro-lycopene [42]. Pro-lycopene is then isomerized by a specific carotenoid isomerase (CRTISO) into all-trans-lycopene. At this level, the pathway is divided into two branches. A first branch leads to the formation of β-carotene and its xanthophyll derivatives, such as zeaxanthin and ASX, and the second branch leads to the formation of α-carotene and lutein in microalgae. The formation of β and ε rings at both ends of lycopene results in a yield of α-carotene under the catalysis of lycopene β-cyclase (LCYB) and ε-cyclase (LCYE) respectively, whereas the formation of two β-rings at the two ends of lycopene gives rise to β-carotene catalyzed by LCY-b alone [42]. Β-carotene can be further hydroxylated by the β-carotene hydroxylase (CHYB) to give zeaxanthin. The amounts of carotenoids produced by each branch of the pathway are dependent on activities of LCYE and LCYB. α-carotene hydroxylation is assured by two heme-containing cytochrome P450 monoxygenases (namely, β carotene and carotene ε-hydroxylases), which formslutein. In a first branch, zeaxanthin is transformed into violaxanthin by the enzyme zeaxanthin epoxidase (ZEP), which incorporates two epoxy groups at positions C-5,6 and C-5′,6′. Zeaxanthin is also transformed in a second branch into di-keto carotenoid canthaxanthin, and violaxanthin is converted to ASX by β-carotene ketolase (BKT) (Figure 1). The two pathways zeaxanthin-antheraxanthin-violaxanthin (VAZ pathway) and diadinoxanthine-diatoxanthin (DD-DT pathway) (Figure 1) are reversible and serve to dissipate cellular energy upon exposure to high light. This mechanism of photoprotection is called the xanthophyll cycle [101,102].

## 5. Cellular Location and Function of Xanthophylls

Xanthophylls can be distributed in different ways in cell compartments depending on their structure. They are generally associated with membranes and/or non-covalently bound to specific proteins [103]. According to their functions, carotenoids can be either primary or secondary [4]. Primary carotenoids, particularly violaxanthin, antheraxanthin, zeaxanthin, lutein, neoxanthin and β-carotene, function primarily as light harvesting pigments due to their association with the structural and functional components of the photosynthetic apparatus [4]. In contrast, secondary carotenoids such as ASX and canthaxanthin are mainly produced under well-defined abiotic stress conditions to protect cells against oxidative stress. Regarding their location, secondary carotenoids are located in the lipid vesicles in the stroma of a plastid or in the cytosol, while primary carotenoids are found in the thylakoid membrane [4]. However, some carotenoids produced in chloroplasts can be exported into the cytoplasm and are therefore found anywhere in cells [104].

Xanthophylls assure various physiological functions in microalgae: they are primarily involved in light harvesting, but also participate to stabilizing the structure and aid in the function of photosynthetic complexes besides protecting chlorophyll from being damaged by visible light or near-UV radiation [105]. They safeguard unsaturated fatty acids (UFAs) contained in the cellular membrane from photo- and pero-oxidations [103]. Furthermore, they act as efficient antioxidants by scavenging free radicals, which may attack DNA and RNA, as well as metabolites such as membrane proteins [103].

## 6. Recent Applications in Metabolic Engineering for Xanthophylls Production

Microalgae are considered to be ideal model hosts for metabolic engineering since they offer many advantages, such as a simplicity of culture and fast growth rates compared with plants. Furthermore, eukaryotic microalgae present more genetic and physiological similarities with plant cells than bacteria [96]. In addition, many microalgae have an active central terpenoid metabolism, allowing a great enough supply of precursors and a high capacity for xanthophylls storage [96]. Nevertheless, studies into the modification of these pigment pathways in microalgae are infrequent.

Mutagenesis, with its various methods, has been applied to wild strains of microalgae to improve their xanthophyll production. In 2001, Jin and collaborators used mutagenesis to increase production of zeaxanthin by *Dunaliella salina* and ultimately succeeded in generating two overproducing strains of zeaxanthin. One of these zeaxanthin epoxidase mutants was recognized in the study carried out by Jin and Melis in 2003 [106]. Similar mutations have also been conducted in other microalgae strains, such as *Scenedesmus obliquus* and *Chlamydomonas reinhardtii* [107]. The zeaxanthin content (per cell) is 15-fold higher than the wild type under non-stressed conditions [108]. Zeaxanthin has previously been engineered by chemical mutagenesis into *Chlorella zofingiensis* [109]. Its production reached 4 and 7 mg/g DW in mixotrophic and photosynthetic conditions, respectively [109]. Manipulation of microalgae to make them able to produce higher quantities of xanthophylls can also been carried out by the inactivation or the overexpression of own genes or by the expression of genes from other species [16]. This would allow us to obtain genetically modified species of microalgae with ameliorated or decreased enzymatic activities and able to accumulate the desired pigments. Recently, in 2018, Sarnaik et al. introduced the exogenous β-carotene oxygenase gene (CrtR) from the strain *Synechoccocus elongatus* (PCC 7002), to prolong carotenoid pathway toward zeaxanthin production in vivo [110]. Associating the CrtR gene with the insertion of the galactose transporter from *Escherichia coli* led to respective zeaxanthin yields of 9 mg/g DW and 8 mg/g DW in autotrophic and mixotrophic conditions [110]. ASX is commercially produced, generally for its human health benefits, and is used as an anti-inflammatory compound and as a skin protector. It can also be used as an essential component of aquaculture feed [71,111]. *Haematococcus pluvialis*, the most common strain of microalgae, used for the production of ASX, accumulates between 9 and 36 mg/g DW of this pigment depending on culture conditions. However, production of ASX by other species is currently under study to bypass the constraints of culture observed with *Haematococcus* strains [112]. Even if *Synechocystis* sp., a cyanobacteria, does not naturally express the genes encoding for BKT and CRTR-B, and the enzymes can be engineered using genes from *Haematococcus pluvialis* under the promoters cpc560 for BKT and psbA2 CrtR-B [113]. These successful gene expressions led to the conversion of ASX precursors echinenone and zeaxanthin, into 4.8 mg/g DW ASX in nitrogen deprivation [113]. Similarly, the *Chlamydomonas reinhardtii* UVM4 strain mutant has become able to produce ASX after a redesign by codon optimization of the Bkt gene. The latter was a dysfunctional endogenous gene [114] which was targeted at the chloroplast with a psaD transit sequence. Hence, ASX production yields of 1 and 3 mg/L/day in autotrophy and mixotrophy conditions were respectively achieved [114]. Additionally, the endogenous PDS was recently overexpressed in the chloroplast of *Haematococcus pluvialis* by optimizing codons and using the psbA promoter and UTR [115], demonstrating the capacity of expressing a nuclear gene successfully in *Haematococcus pluvialis* chloroplast. ASX yields reached in the efficient transformants were up to 34 mg/L instead of 18 mg/L in the wild strain microalgae [115]. Fucoxanthin is another highly researched carotenoid due to its anti-obesity, anti-diabetic and anti-cancer properties. It is also well established as strong antioxidant compounds [29]. With the aim of increasing fucoxanthin production, Manfellotto et al. (2020) transformed *Phaeodactylum tricornutum* with single plasmids or combinations of them for the overexpression of genes with a putative role in xanthophylls biosynthesis [116]. They obtained two triple transformant genes: Vde-related (VDR),Zeaxanthin epoxidase 3 (ZEP3) and Violaxanthin de-epoxidase (VDE), were over expressed, allowing the carotenoids accumulation with a four-fold increase in the fucoxanthin content compared to the wild strain [116]. Similarly, overexpression of the Psy gene in *Phaeodactylum tricornutum* allowed a fucoxanthin content 1.45 times higher than that in the wild type strain [117]. In the same way, overexpression of Dxs and Psy genes led to an increase in fucoxanthin content of 2.4 fold and 1.8-fold respectively [117,118]. Similar metabolic engineering studies were realized for the over production of lutein. 

*Chlamydomonas reinhardtii* was transformed using the genes of *Dunaliella salina* and *Chlorella zofingiensis*, producing respectively 2.6- and 2.2-fold higher yields of lutein [119]. A point mutation was introduced in the endogenous gene encoding PDS of *Chlamydomonas reinhardtii* to enhance its expression. Concomitantly to it, the increase of lutein production was observed [120]. In a very similar approach using *Chlamydomonas reinhardtii*, the gene encoding for PBS from *Xanthophyllomyces dendrorhous* was introduced into pMS188 plasmid and the nuclear transformed [121]. The mutated microalga possesses a bifunctional enzyme with the two PSY and LCYB activities. This allowed carotenoid accumulation for the first time using an heterologous expression system, leading to a simultaneous increase of about 60% in lutein biosynthesis under low light culture conditions. Random mutagenesis has been effectively used for the production of *Chlorella sorokiniana* mutants with high contents of lutein [119]. 

In another study, ethyl methane sulfonate and N-methyl-N′-nitro-N-nitrosoguanidine have been used as chemical mutagenes for generating a lutein-deficient *Chlorella vulgaris* (CvLD), which was found to be an enhanced producer of the pigment violaxanthin [55]. The sequencing of the lcy-e gene of this lutein-deficient *Chlorella vulgaris* led to the identification of a single mutation at the position 336. The mutated Valine, instead of an Alanine, might have occurred in the active site of the lycopene ε-cyclase, decreasing its activity.

## 7. Bioprocess for Xanthophylls Production by Microalgae

To acquire high xanthophyll productivity, both biomass production and its pigment content need to be optimized. 

### 7.1. Cultivation Systems 

At this time, xanthophyll production from microalgae is achieved in open pond systems or the closed photo-bioreactors (PBRs).

#### 7.1.1. Open Systems

The cost of construction and operation in open systems are reported to be much lower than for closed PBRs and the cultivation process is also simpler. Open ponds is the most commercially used method for cultures of microalgae, in which the medium flow occurs through a system of paddle-wheels. The latter keeps the cells in suspension and provides better mass transfer [122]. The low deepness in these open systems ensures the light penetration efficiency. The system flow is continuous, so nutrients are continuously supplied and microalgae are harvested at the same time [123]. This system is ideal for the growth of microorganisms that tolerate and can grow under extreme environment conditions such as high alkalinity (*Spirulina* sp.), high salinity (*Dunaliella salina*) and nutrient-rich media (*Chlorella* sp.). Indeed, the photoautotrophic culture mode has been extensively employed for *Dunaliella* with the aim of carotenoid overproduction [124]. A two-step system, namely “intensive cultivation”, allowed large-scale carotenoid production in *Dunaliella*. The aim of stage one is to promote biomass accumulation with a weak β-carotene-chlorophyll ratio; and in stage two, *Dunaliella* culture is diluted three times to increase the light penetration to cells, and carotenogenesis after nitrogen depletion [79]. Therefore, it is always recommended to use open and raceway ponds for cultivating microalgae using a photoautotrophic growing condition to minimize contamination issues. However, microalgae that cannot grow in these specific environmental conditions, such as *Tetraselmis* sp., *Isochrysis* sp., *Crypthecodinium* sp., and *Skeletonema* sp., should not be cultured with this type of approach [125]. Some drawbacks are observed from this method; for example, it is difficult to control the conditions around the tank, such as the temperature and light, and there is a high risk of contamination by other algal/bacterial strains [126]. Other crucial factors could have a great influence on these systems amongst other large losses of water after evaporation, CO_2_ diffusion into the atmosphere, and the need for large land areas [122]. Therefore, the closed systems seem to be preferred. 

#### 7.1.2. Closed Systems

Open system problems have led to the design of closed systems. PBRs represent the most successful approach to attain better control of important culture parameters like pH, light, temperature, loss of H_2_O, capture of CO_2_ and biomass productivity [127]. Furthermore, the low contamination risk is a main asset that would permit a higher control and production of molecules with high-commercial values, such as xanthophylls. PBRs were designed for the cultivation of microalgae [123,126], including the following types:

- The tubular type is the most appropriate kind of PBR for producing satisfying high-quality cyanobacteria and microalgae biomasses in outdoor environments [123,128]. It is generally built with glass or plastic tubes, allowing a large illuminated surface area. In this system, the culture homogenization is generally assured by means of air pumps. It is characterized by some defects, such as pH variation, dissolved oxygen, fouling, and CO_2_ heterogeneity. There are many studies indicating the suitability of using this PBR kind for high-quality microalgae and cyanobacteria productions.
- Flat PBRs have a large surface exposed to light and are characterized by high algal productivities, which is generally greater than those produced by tubular PBR. This culture system is constructed from a rigid transparent material to optimize light capture and to facilitate sterilization. It is suitable for outdoor cultivation, ideal for cell immobilization and is relatively inexpensive. The only drawback of this type of system is the difficulty in controlling the temperature of algal cultures [129]. Flat PBRs have been tested for culturing the marine diatom *Phaeodactylum tricornutum* for the production of fucoxanthin and chrysolaminarin [130]. The AlgaTechnologies industry (https://www.algatech.com/, accessed on 15 April 2021) also established a *Haematococcus* cultivation facility back in the late 1990s. Quite different to other American industries, the AlgaTechnologies Company used glass tubular PBRs for both green and red phases to phototrophically cultivate *Haematococcus* [131].

Problems that are associated with a limited light source that hinders high cell density in large-scale PBRs during photoautotrophic growth can be avoided by using heterotrophic cultivations [132]. The elimination of light restriction led to a higher microalgae cell growth rate and a greater cell mass content can be reached faster. So far, the maximum produced biomass in photoautotrophic conditions was about 40 g/L of microalga dry weight [133] and this content was lower than in that obtained in heterotrophic conditions (150 g/L) [134]. Heterotrophic culture mode is mainly used for the production of high value-added xanthophylls (lutein, ASX…) from microalgae, due to its high cost [79]. The dry weight of the green microalgae *Chlorella protothecoides* and its lutein content attained 19 g/L and 84 mg/L respectively, when it was cultured heterotrophically. These values reached 47 g/L and 225 mg/L using a culture with the fed-batch system [135]. Wu and Shi (2006) found the highest biomass concentrations and a maximum productivity of 105 g/L cell dry weight and 0.613 g/L/h, respectively, when *Chlorella pyrenoidosa* was grown in heterotrophic conditions [136]. 

Hybrid systems are deployed for the large-scale production of marine microalgae to produce some molecules of commercial interest. In 2015, The diatom *Staurosina* sp. and the chlorophyte *Desmodesmus* sp. were cultured in a hybrid system combining PBRs (25 m^3^) and open basins (400 m^2^) [137]. In this system, the PBRs permanently ensured a source of uncontaminated inoculum for the short-lived batch culture in an open pond. The latter ensures a large-scale biomass production in a competitive cost- and time-consuming operation. For the production of ASX from the green microalgae *Haematococcus pluvialis*, Cyanotech corporation https://www.cyanotech.com/is, (accessed on 15 April 2021) an example of industries that use PBRs for the green phase (vegetative growth) and ponds as open systems for the red phase (production of xanthophylls). Mera Pharmaceuticals Inc. (http://www.merapharma.com/, accessed on 15 April 2021) was among the first companies which established large scale ASX production facilities globally. The company was located where weather conditions are extremely suitable for outdoor *Haematococcus* cultivation [138]. Based on the same system, the company employed a two-step autotrophic cultivation approach. The green phase of microalgae growth was conducted in 25,000 L computerized outdoor PBRs, and the red phase of ASX accumulation in raceway ponds. Mixotrophic culture of *Haematococcus* has long been sought as an alternative approach for the traditional two-step ASX production process [139,140]. In fact, the world’s first commercial ASX production facility from the microalga *Haematococcus* appears to have been based on mixotrophic culture technologies in 1995 [138]. However, since then, phototrophic culture approaches have been amply developed and have become the main strategy for the production of ASX from microalgae. The advantage of mixotrophic cultivation is that the production can be carried out indoors and under optimal controlled conditions. However, the cost of materials and energy consumption might be too high to compete with phototrophic culture using sunlight. Recently, a novel ASX production process based on a mixotrophic mode has been developed with a heuristic multilevel LED light regime and the highest content of 3.3% was achieved at the white-blue regime [141]. Polyol alcohols (glycerol and mannitol) have also been shown to be more efficient carbon sources than acetate for the efficient and cost-effective ASX production from *Haematococcus* [142].

### 7.2. Factors Determining Xanthophylls Production

Secondary xanthophylls production is monitored by changes in culture conditions and different stress factors [143]. Xanthophyll production is improved by ROS, under stress conditions like high light intensity and salinity [144]. For this reason, ASX is assumed to protect organisms from free radical-linked diseases like cancer [145]. Several disadvantageous environmental conditions like nutrient deprivation, excessive photosynthesis and extreme irradiationreduce the incidence of electron transfer and, therefore, photo-oxidative damage [146]. Primary xanthophylls, like lutein, deteriorate under stress conditions, hence their content in cell biomass is diminished. Combined effects of numerous stress factors have ameliorated the ASX production in many microalgae such as *Haematococcus pluvialis* [147].

#### 7.2.1. Light

Light availability constitutes the most effective controlling factor for the production of numerous xanthophylls [119]. Light intensity and photoperiods affect the growth of cells, biomass and production of several high-value metabolites in many microalgae species. Higher light intensity caused a threefold increase in *Haematococcus pluvialis* ASX content. Besides, ASX and lutein contents are also changed under a high intensity of light [148]. *Muriellopsis* sp. lutein content reached the maximum at 460 µmol photons/m^2^/s. Maximum lutein productivity (3.6 mg/L/day) was obtained under high light intensity with *Desmodesmus* sp. [149] The lutein synthesis and accumulation were studied in *Chlorella sorokiniana* [119] and *Scenedesmus* sp. [78] because they both have high growth rates and high lutein production ability. Light stress was applied concomitantly with other stressors such as nitrogen [150], salinity [151] and temperature [152]. In addition, Zhao et al. (2018) demonstrated that of nitrogen lack and light stress together enhanced the ASX accumulation in *Haematococcus pluvialis*, which reached 1.85% of the cell dry weight [153]. Light stress can stimulate the expression of the lycopene beta-cyclase gene [154], which is the key enzyme for carotenoid accumulation in microalgae. Moreover, Coesel et al. (2008) also showed that high light intensity can regulate the activities of phytoene synthase and phytoene desaturase [155].

#### 7.2.2. Temperature

High temperatures play an important role in the accumulation of xanthophylls in microalgae due to the photooxidative stress [40]. High temperatures affect the synthesis of ASX in *Haematococcus pluvialis* [156] and *Chromochloris zofingiensis* [40]. Indeed, temperatures above 28 °C minimize the total productivity of ASX in *Chromochloris zofingiensis* [148] because the ROSs are more assimilated through photosynthesis, which stimulates the buildup of ASX [157]. High temperatures also result in the accumulation of lutein in *Dunaliella salina* [158]. Similar observations were found for *Chlorella protothecoides* strains [135]. However, low temperatures decrease the nutrient uptake rate and slow the lutein accumulation [159].

#### 7.2.3. Salinity

The salinity effect on microalgae growth and xanthophyll formation is complex. Many microalgae species are able to tolerate high salinity levels because of their osmoregulation capacity, which involves constant glycerol synthesis [160]. Indeed, salt stress has a positive effect on the production of secondary xanthophylls such as ASX. For example, an ASX content of 5 mg/g was obtained with *Haematococcus pluvialis* when treated with 0.2 g/L of NaCl, which was 42% higher than that of the control [161]. A general increase in ASX production was also observed with *Chlorella zofingiensis* grown at NaCl concentrations of up to 0.2 M [148]. Furthermore, salinity stress alone did not change the content of lutein in *Dunaliella salina* [162], contrary to the combination of nitrate concentration and salinity [163]. 

#### 7.2.4. Nutrient-Related Stresses

##### Nitrogen Starvation

The concentration of nitrogen in the culture medium and xanthophyll content of microalgae were correlated in many species [164]. The production of xanthophylls was increased by nitrogen limitation in *Neochloris oleoabundans*, *Chlorella zofingiensis, Dunaliella salina*, and *Muriellopsis* sp. [25,165,166]. In addition to ASX and lutein, β-carotene level was enhanced to 2.7% DW in *Dunaliella salina* cells in nitrogen-depletion [167]. Under the same conditions, xanthophylls, namely ASX and canthaxanthin, initiate their accumulation in aerial microalgae (*Coelastrella* sp.); after this, the color of the cells changes from green to red [56]. Nitrogen starvation can promote the concomitant accumulation of ASX in microalgae [120,168] as well as lutein accumulation. The latter is highly dependent on nitrogen concentration in the culture medium [165]. In general, nitrogen deficiency has a greater impact than excess nitrogen on carotenoid production, mainly that of ASX in *Haematococcus pluvialis*, because a culture growing in a nitrogen-rich medium requires carbon to assimilate the nitrogen. On the other hand, high competition for carbon required for xanthophylls synthesis would be established in low nitrogen concentrations [169]. A mixture of urea and other nitrogen sources has led to a maximal lutein production in *Auxenochlorella protothecoides* [77]. A two-step mode where nitrogen enriched and nitrogen deficient media were used consecutively stimulated the microalgae growth during the first phase and carotenoid enrichment during the second phase. 

##### Iron Supplementation

Iron is needed for microalgae growth such as *Dunaliella*. Of all the micronutrients, iron was the best for accumulation of ASX in *Haematococcus pluvialis* cysts [144] because iron acts as a chelating agent and can scavenge hydroxyl radicals in the Fenton reaction, which is widely used in the enzyme system of animals, microbes, and plants [170]. It acts as a limiting factor under hyper saline conditions. The site for assimilation of iron is usually the plasma membrane [171]. He et al. (2007) reported that ASX accumulation depends on many nutrients, such as phosphorus, iron, and sulfur [171]. However, adding Fe^2+^- EDTA to culture medium produced ASX up to 3.1% dry cell in *Haematococcus pluvialis* [172].

##### Sulfur Limitation

Sulfur limitation is beneficial for xanthophylls production by microalgae and its starvation is more efficient than that of iron for high level accumulation of ASX [173]. 

Sulfur is essential for the glutathione biosynthesis [174]. Glutathione acts in the oxidative stress response as a ROS scavenger. So, an increase of ROS caused by sulfur limitation led to decrease of GSH concentration and can enhance the carotenoids production. Additionally, many authors suggested that glutathione might act as a “sensor” of the cell’s sulfur status, thus regulating the rate of sulfur assimilation [174,175].

## 8. Encapsulation of Xanthophylls

As summarized in Table 3, numerous attempts have been made concerning the encapsulation of xanthophylls from microalgae. Machado et al. (2014 and 2016) proved that an aqueous fluid of andrographolide made by particle engineering Supercritical (SEDS) is a promising approach for the encapsulation of ASX isolated from *Haematococcus pluvialis* [176,177]. ASX encapsulation based on a co-precipitation of PHBV with supercritical CO_2_ and DCM respectively as an anti-solvent organic solvent was studied by Machado et al. (2014 and 2016) [176,177]. Mean particle sizes of 0.128 μm was achieved with the a maximum Presure = 100 bars [176]. In addition, at the carotenoid extraction phase, a maximum of encapsulation efficiency (EE) of 48.25% was realized utilizing [B/DCM] = 10 mg/mL. At [B/DCM] = 10 mg/mL and 80 bars, globular drops with 0.228 μm were gained. Overall, a pressure extension caused a reduction in EE [177]. By using a spraying technique, Park et al. in 2014 reviewed the EE of ASX-extracted from *Xanthophyllomyces dendrorhous*. These authors confirmed that the microparticles varied from 10 to 800 μm, with an average of 210.26 μm and EE was within 68 and 79% [178]. Bustos-Garza et al. (2013) studied the pH-stability and thermal properties of ASX encapsulated by spray drying [179]. These authors used gum Arabica (GA) and whey protein (WP) individually or in association with maltodextrin (MD) or inulin (IN) as wall materials, and established circular micro particles, with a size that ranged between 1 and 10 μm. In another study, Higüera-Ciapara et al. (2004) examined the ASX/CT matrix microencapsulation [180]. The fabricated product (microcapsules) had a Ø of 5–50 μm. In a study by Kittikaiwan et al. (2007), encapsulated ASX was evaluated against oxidative stress [181]. *Haematococcus pluvialis* was entrapped into beads, which were then coated with 5 layers of CT film, resulting in CT-algae capsules that have a Ø of 0.43 cm and the thickness of the film was ~100 μm. By precipitation processes using supercritical fluid (200 bars and 35 °C), Hong et al. (2009) studied *H. pluvialis* ASX, and obtained particles with Ø of 0.5 and 3 μm [182]. Similar findings were obtained by Tachaprutinun et al. (2009) [183].

By using an external ionic gelation technique, Niizawa et al. (2019) evaluated the effect of five independent formulations (Tconcentrations of: CaCl_2_, oleoresin, alginate/oleoresin, alginate and surfactant) for natural ASX oleoresin encapsulation [184]. Mathematical models were developed to predict size of particle, ASX t1/2 release and EE. If oleoresin-enriched beads Ø were linked to alginate and alginate/oleoresin levels, EE was mannered by surfactant and alginate concentrations. These parameters have an impact on the kinetic modeling on ASX release under an intestinal micro bioassay. Lin et al. (2016) reported the same results [185].

According to Boonlao et al. (2020), ASX-enriched O/W emulsion was controlled by WPI (2–5 wt %) and XG (0.25 and 0.5 wt %) [186]. Compared to blends supported by WPI, XG addition increased the stability of the emulsion. The ASX enclosed in an WPI-XG structure was more constant, at 5, 25 and 37 °C. Through simulated digestion, WPI-XG trials showed a lesser globule dimension inside the gastric and intestinal stage, demonstrating that the XG enhances the emulsion. XG blended with WPI established lesser lipid digestibility and restricted the free fatty acid composition.

Through nanoencapsulation, Zanoni et al. (2019) developed a method to stabilize the ASX of *Haematococcus pluvialis* to improve its nutritional properties and to increase its bioavailability [187]. Nanoparticles (NPs) were prepared by an emulsification–solvent evaporation technique, and oleoresin at 1%. At this concentration (1%), NP Ø was equal to 90 nm. Regarding NPs Zeta-potential (ζ potential), values were due to the WP covering that is negatively charged at a neutral pH. The stability of the NPs was examined through a panel of stress experiments (Fe^3+^ exposition, heat at 65 °C, extreme pH and UV radiation, and). Simulated gastroenteric digestion was carried out to examine ASX release in physiological terms and was presented at a high bioaccessibility (76%).

In another study, Zhou et al. (2018) made, through electrostatic complexation of WP and GA by adjusting the pH to 4.0, esterified ASX microcapsules [188]. Biochemical characteristics of the esterified ASX microcapsules were evaluated, and the gastrointestinal potential fate and bioavailability were observed through in vivo and in vitro digestion trials. At a stabilized system, Ø was equal to 15.4 μm, and EE was 95.3%. For in vivo experiments, after breads oral gavage, the area under the curve (AUC0-t) was 8.23 h·μg/mL and was twofold greater than those of oleoresin (3.72 h·μg/mL) [188].

By using the vibrating nozzle technology, ASX-enriched oil was encapsulated in alginate and low-methoxyl pectin [189]. Authors studied the ASX degradation kinetics by fitting the data with deferred zero-, first- and second-order kinetic models. Interestingly, low methoxyl pectin exposed the appropriate ASX-enriched oil encapsulation. Previously studied conducted by Pu et al. (2011) [190], Niamnuy et al. (2008) [191] and Takeungwongtrakul and Benjakul (2016) [192] and Bustamante et al. (2016) [193] selected the degradation of ASX as a first order reaction.

## 9. Conclusions and Perspectives

By virtue of their importance to the food industry and human health, xanthophylls produced by microalgae have been extensively studied in the past two decades. Numerous studies have been led to evaluate the efficiency of several conventional and innovative extraction techniques for the isolation of various xanthophylls from diverse species of microalgae. Many challenges still remain, as it is necessary to combine nonthermal processing technologies to achieve sustainable processing and assure safe outputs, which may offer a new way to obtain xanthophylls with high quality. More researches are needed to provide “environmentally friendly processes”.

Enzymes and genes of the biosynthetic pathway of xanthophylls have been widely investigated. Nevertheless, regulation remains to be completely elucidated. Functional studies of identified putative xanthophyll regulators of the several species are mandatory to increase a deeper study of their metabolism. Equally, promotor examination will be supportive for the identification of novel transcriptional regulators of late xanthophylls biosynthetic pathway genes. In this regard, integrative analysis of multi-omics data such as genomics, transcriptomics, metabolomics and proteomics will allow us to have a better understanding of the expression profiles of xanthophyll biosynthetic pathway genes in microalgae.

On the other hand, future research into newer capsules should also command attention regarding the widening range of hues that can be gained, and on promoting the xanthophylls with linked health-beneficial features. Further studies are required on xanthophylls stabilization, which, to date, has been treated using diverse methods founded on encapsulation. 

## Figures and Tables

**Table 1:** Xanthophylls from microalgae: mass production, extraction method and application.

Xanthophylls	Microalgae	Extraction Processes	Concentrations	Applications	References
Fucoxanthin	*Tisochrysis lutea*	Ultrasonic-assisted extraction	0.25 mg/g dw	Nutraceutical, cosmetic and pharmaceutical applications	[26]
	*Cyclotella meneghiniana*		2.3 mg/g		[27]
	*Mallomonas* sp.		26.6 mg/g		
	*Nitzschia* cf. *carinospeciosa*		5.5 mg/g		
	*Phaeodactylum tricornutum*		10 mg/g		
	*Paralia longispina*		1.4 mg/g		
	*Isochrysis* aff. *galbana*		1.8% dw		[28]
	*Odontella aurita*		up to 2.2% dw		[29]
	*Thalassiosira weissflogii*	Solvent extraction	5.1 mg/L/d		[30]
ASX	*Haematococcus pluvialis*	Conventional extraction	900 kg/2 ha/year	Antioxidant, anti-cancer, anti-inflammatory, ocular protective effect, antidiabetic, coloring agent	[31]
		Two-stage system	3.8% dw		[32]
		Enzyme extraction	3.8% dw		[33]
		Conventional extraction	2–3% dw		[34]
		Pressurized extraction	99% of total AS		[35]
	*Haematococcus lacustris*	Mechanical extraction	18.8 mg/L		[36]
Lutein	*Chlorella vulgaris*	Heptane–ethanol– water extraction	30 mg/g	Antioxidant, light-filtering, eye protection, colorant, potential therapeutic use against several chronic diseases, lower risk of cancer, anti-inflammatory benefits	[37]
	*Chlorella minutissima*	Solvent extraction	5.58 mg/g		[38]
	*Chlorella sorokiniana*	Solvent extraction	7.62 mg/L/d		[39]
	*Scenedesmus bijugus*		2.9 mg/g		[40]
	*Dunaliella salina*	Conventional extraction	15.4 mg/m^2^/d		[41]
	*Chlorella protothecoides*	Maceration	83.8 mg/L		[42]
	*Chlorella protothecoides*	Mechanical	4.92 mg/g		[43]
	*Tetraselmis* sp. CTP4	Mechanical	3.17 mg/g dw		[44]
	*Chlamydomonas* sp.	Solvent extraction	5.08 mg/L/d		[45]
	*Muriellopsis* sp.	Solvent extraction	100 mg/m^2^/d		[46]
	*Chlamydomonas acidophila*	Solvent extraction	20 mg/L		[47]
	*Scenedesmus almeriensis*	Accelerated solvent extraction	8.54 mg/g		[48]
	*Scenedesmus obliquus*	Solvent extraction	3.63 mg/g		[49]
	*Desmodesmus* sp.	Solvent extraction	5.22 mg/L/d		[50]
	*Coelastrella* sp.	Accelerated solvent extraction	6.49 mg/g		[40]
Zeaxanthin	*Heterochlorella luteoviridis*	Moderate electric field	244 µg/g	Antioxidant, anti-inflammatory, eyes and UV light protection, prevention of coronary syndromes, anti-tumoral, anti-cardiovascular diseases, and structural actions in neural tissue	[21]
	*Nannochloropsis oculata*	Supercritical fluids extraction	13.17 mg/g		[51]
	*Chlorella ellipsoidea*	Pressurized liquid extraction	4.26 mg/g		[52]
	*Synechocystis* sp.	Pulse electric field	1.64 mg/g		[53]
Violaxanthin	*Chlorella ellipsodea*	Solvent extraction	not determined	Anti-inflammatory activity	[54]
	*Chlorella vulgaris*	Solvent extraction Mechanical extraction	3.7 mg/g		[55]
Canthaxanthin	*Coelastrella striolata* var. *multistriata*		4.75% dw	Anti-oxidant property Create a tan color	[56]
	*Chlorella vulgaris*		45% Total carotenoids		[57]
Cryptoxanthin	*Spirulina platensis*	Supercritical fluid extraction	7.5 mg/100 g	Antioxidant, anti-inflammatory, anticancer, improves respiratory functions, stimulates bone formation and protection, decreases risk of degenerative diseases	[58]
	*Pandorina morum*	Maceration	2.38 µg/g DW		[59]
	*Nanochlorum eucaryotum*	Enzyme extraction	not determined		[60]
Diadinoxanthin	*Odontella aurita*	Ethanol extraction	10% total carotenoids	Antioxidant	[29]
	*Phaeodactylum tricornutum*		19% of total pigments		[61]
Diatoxanthin	*Phaeodactylum tricornutum*	Methanol extraction	17% of total pigments	Antioxidant	[61]

**Table 3:** Main examples of encapsulated microalgal astaxanthin and fucoxanthin.

Bioactive Compounds	Wall Materials	Encapsulation Techniques	Encapsulation Preparations	Main Findings	References
ASX from *Haematococcus pluvialis*	PHBV (Poly(hydroxybutyrate-co-hydroxyvalerate))	Co-precipitation	- PHBV was used at 20 mg/mL in an organic solution (T = 35 °C, Flow rate = 1 mL/min) - The precipitation pressures were 80 and 100 bars.	- EE = 51.21% at high ratio biomass/DCM, and lower pressure of precipitation (80 bars). - Ø = 0.228 μm	[176]
ASX from *Haematococcus pluvialis*	PHBV (Poly(hydroxybutyrate-co-hydroxyvalerate))	Co-precipitation	- PHBV was used at 20 mg/mL in an organic solution - The precipitation pressures were 80 and 100 bars.	- At 100 bars, Ø = 0.128 μm, - EE = 48.25%	[177]
ASX from *Haematococcus pluvialis*	Precirol ATO 5 or Stearic acid	Hot Homogenization method: SUPRAS/NLCs mixture	- Aqueous phase (AP): At 65 °C Poloxamer 188 or 407 in 15 mL of water. - Oil phase (OP): At 65 °C, Precirol ATO 5 or stearic acid, soy lecithin and 750 µL of SUPRAS were heated. - AP was included in AP - Centrifugation (13 min at 13,000 rpm) - Recover of SUPRAS-NLCs	- EE = 71% - Ø∼100 nm. - ASX antioxidant activity waas preserved	[87]
ASX from *Haematococcus pluvialis*	GA and WP single or mixed with MD or IN	Spray drying	- Aspirator air flow rate = 32.9 m^3^/h - *p* = 40 kg/cm^2^, T = 25 °C - Inlet and outlet air T = 120 and 70 °C, respectively.	- EE (WP) = 61.2% - EE(GA) = 70.1%. - The capsules displayed red and yellow colors - As regards h values: GA–WP 50:50 (41.2°) < GA–IN 25:75 (42.4°) < GA–IN 50:50 (43.8°) < GA–WP 25:75 (45.2°) < WP (45.8°) < GA (48.0°) < GA–MD 25:75 (61.1°) < GA–MD 50:50 (70.2°). - First-order reaction kinetics (degradation and antioxidant activity). - Established WP particles have higher stability T, - Rank and pH stability: 6 > 5 > 4 > 7 > 3	[179]
ASX from *Haematococcus pluvialis*	WPC	Emulsification–solvent Evaporation	- Solublization of WPC in water (1–10%) - Dilution of oleoresin was diluted in EtOAc (1–11%) and blended with WPC (9:1) - Production of emulsion by an ultrasonicator (10 min, 10 W).	- EE = 96%. - Ø (80–130 nm) - ζ potential (−20 and −30 mV). - NPs precipitation at pH 3.5–5.5. - ASX high bioaccessibility = 76%.	[187]
Esterified ASX from *Haematococcus pluvialis*	WP and GA	Complex coacervation	- WP and GA solutions at 2.0% were prepared within 0.2 M PO_4_^2−^ buffer (pH 7.0). - OP: preparation of 20% (*w/w*) of esterified ASX oleoresin in corn oil - For microcapsule fabrication, 3 g of OP was distributed into WP - *p* = 400 bars, T = 40 °C. - Blend system, with GA, was agitated (30 min at 700 rpm).	- High stablity of esterified ASXs. - *In vitro* experiment: rate of ASX and oleoresin release from the microcapsules were 26% and 14.6%.	[188]
ASX-enriched oil from *Haematococcus pluvialis*	C6H7NaO6; and low-methoxyl pectin	Vibrating nozzle technology	- T = 40 °C - *p* = 500 mbars, and 600 Hz, 2000 V, 3A. - Ø nozzle = 750 µm.	- Then 52 weeks, total-ASX retention = 94.1% with various degradation kinetics.	[189]
Fucoxanthin from *Chaetoceros calcitrans*	Maltodextrin and GA	Spray and freeze drying	- Freeze-drying T = at −80 °C during 24 h *p* = 25 × 10^−2^ Pa, T = −40 °C - Spray-drying *p* = 6.5 bar at 8.5 mL/min. Air flow = 30 m^3^/h, inlet/outlet T of 100/70 °C.	- Well behaviors of fabricated particules into within food powder - antioxidant activity was preserved	[194]
Fucoxanthin from *Phaeodactylum tricornutum (FX)*	Chitosan (CN)	Electrospraying	- V = 5 kV, Flow rate = 130 μL/min. - T = −120 °C during 48 h.	- EE = 71%, polydispersity index = 0.31–0.39 in H_2_O. - ζ potential of FX-CS (casein)-CN and FX-CN were 24.00 and −12.87 mV, respectively. - FX bioaccessibility > FX-CN > FX-CS-CN. - In C57BL/6 mice, fucoxanthinol absorption to the blood circulation was two times higher for FX-CS-CN versus FX-CN.	[195]

## Data Availability

The data are included in the manuscript.
